# Association of phthalate metabolites with periodontitis: a population-based study

**DOI:** 10.1186/s12903-024-04316-4

**Published:** 2024-05-08

**Authors:** Mengyao Bian, Wenxiang Jiang, Manting Wang, Ying Shi, Zhifang Wu

**Affiliations:** Stomatology Hospital, School of Stomatology, Zhejiang University School of Medicine, Zhejiang Provincial Clinical Research Center for Oral Diseases, Key Laboratory of Oral Biomedical Research of Zhejiang Province, Cancer Center of Zhejiang University, Engineering Research Center of Oral Biomaterials and Devices of Zhejiang Province, Hangzhou, 310000 China

**Keywords:** Phthalate, Periodontitis, NHANES, Epidemiology

## Abstract

**Background:**

Widespread exposure to phthalates may raise the probability of various diseases. However, the association of phthalate metabolites with periodontitis remains unclear.

**Methods:**

Totally 3402 participants from the National Health and Nutrition Examination Survey (NHANES) 2009 to 2014 cycles were enrolled in the cross-sectional investigation. We utilized weighted logistic regression to evaluate the association of ten phthalate metabolites with periodontitis. Restricted cubic spline analysis was applied to investigate potential nonlinear relationships.

**Results:**

The weighted prevalence of periodontitis in the study was 42.37%. A one standard deviation (SD) rise in log-transformed levels of mono-2-ethyl-5-carboxypenty phthalate (MECPP), mono-n-butyl phthalate (MnBP), mono-(2-ethyl-5-hydroxyhexyl) phthalate (MEHHP), mono-isobutyl phthalate (MiBP), mono-(2-ethyl-5-oxohexyl) phthalate (MEOHP), and mono-benzyl phthalate (MBzP) was associated with higher odds of periodontitis, with odds ratios (95% confidence intervals) of 1.08 (1.02-1.14), 1.07 (1.02-1.11), 1.10 (1.05-1.15), 1.05 (1.01-1.09), 1.09 (1.04-1.14), and 1.08 (1.03-1.13), respectively. Individuals with the highest quartile concentrations of MECPP, MnBP, MEHHP, MEOHP, and MBzP were associated with 32%, 20%, 30%, 25%, and 26% increased odds of periodontitis, respectively, compared to those with the lowest quartile. Additionally, mono-(3-carboxypropyl) phthalate (MCPP) demonstrated an interesting inverted J-shaped relationship with periodontitis.

**Conclusions:**

The findings indicate an association of certain phthalate metabolites with periodontitis among US adults.

**Supplementary Information:**

The online version contains supplementary material available at 10.1186/s12903-024-04316-4.

## Introduction

The high prevalence of periodontitis has turned into a major public health issue, burdening the entire world [1, 2]. Notably, periodontitis is known to share a bidirectional relationship with various systemic conditions, suggesting that treating periodontal problems may promote overall health [3, 4]. In recent decades, fundamental research on periodontitis has progressively shed light on the pathogenesis of periodontitis. Current understandings of host-microbe interactions implicate genetics, epigenetics, lifestyle choices, and environmental influences to be critical variables in the onset and progression of periodontitis [5]. Specifically, the establishment of feedforward loops between dysbiosis within complex microbial communities and heightened host inflammatory reactions appear to significantly contribute to the progression of periodontitis [6].

Among environmental contributors, endocrine-disrupting compounds (EDCs) are known to impact human health by altering epigenetic mechanisms [7]. Phthalates, one of the common EDCs, are extensively used in a myriad of products, such as medical supplies, personal care items, food packaging, cosmetics, paints, and toys [8]. Phthalates can be ingested, inhaled, absorbed through the skin, or administered during medical procedures [9]. Existing studies have linked phthalate exposure to numerous health issues, including endocrine system dysfunction, metabolic disorders, infertility, neurologic disorders, respiratory diseases, and cancer [10–12]. The oral cavity, which serves as a common channel of the respiratory and digestive systems, is also susceptible to phthalate exposure. It is questionable whether periodontitis is also associated with phthalate.

A prior study indicated a notable link between mono-n-methyl phthalate and periodontitis [13]. However, the underlying mechanisms are not yet fully understood. The interaction between them may be multi-faceted, involving complex biological processes such as the body's immune-inflammatory responses and oxidative stress [14–16].

Considering the known association of phthalates with various disorders, and given the shared risk factors between periodontitis and systemic diseases [17, 18], we hypothesized that phthalates might be linked to periodontitis. This hypothesis aligns with current understandings of the multifactorial nature of periodontitis, involving both systemic health and environmental exposures. Given the relatively stable presence of phthalate metabolites in the urine across extended periods, they are reliable biomarkers for assessing phthalate exposure [19]. Hence, using cross-sectional data from the National Health and Nutrition Examination Survey (NHANES), we sought to investigate the potential association of urinary phthalate metabolites with periodontitis.

## Methods

### Study design and population

**Fig. 1:**
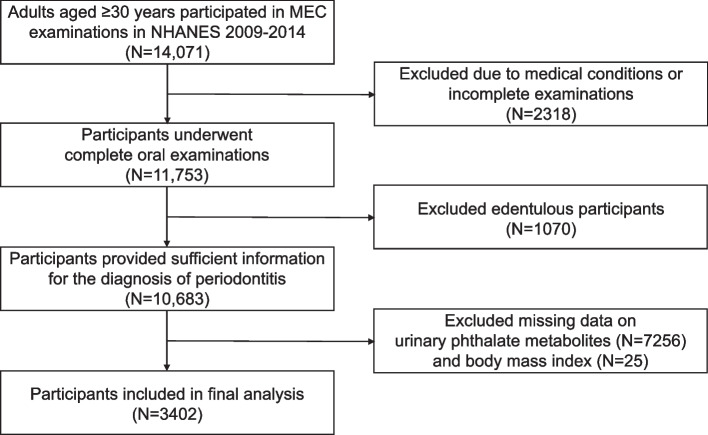
Flow diagram of this study. The final 3402 participants included complete data on urinary phthalate metabolites, urinary creatinine, age, sex, race/ethnicity, and body mass index for calculating the adjusted concentrations of phthalate metabolites. MEC, mobile examination center; NHANES, National Health and Nutrition Examination Survey

The study utilized data from the United States civilian, which was comprehensively surveyed by NHANES through a multi-stage, stratified random sampling methodology. Data on demographics, examination, diet, and laboratory results were collected every two years in this survey. Our cross-sectional study selected data from the NHANES 2009–2014 cycles. Finally, 3402 individuals were selected for inclusion in subsequent analysis. Figure 1 illustrates the process of inclusion and exclusion. The datasets employed for the analyses in this article are publicly accessible for download.

### Periodontitis assessment

Participants aged ≥ 30 years who had a minimum of one natural tooth, were eligible for a full-mouth periodontal examination (FMPE) in NHANES 2009–2014. Licensed dentists used a Hu-Friedy PCP-2 periodontal probe to assess probing pocket depth (PPD) and clinical attachment loss (CAL) at six sites per tooth. Additional information regarding the periodontal examination methodology is detailed in previous studies [20, 21]. For each participant, we calculated the mean probing pocket depth (PPD) and the mean clinical attachment loss (CAL). Periodontitis was classified following the severity criteria set by the Centers for Disease Control and Prevention (CDC) / American Academy of Periodontology (AAP) [22]. For our analysis, we grouped mild, moderate, and severe periodontitis into a single category, termed 'total periodontitis'.

### Phthalate metabolites measurement in urine

Urine samples from the participants were carefully processed, and then stored at -20 °C before being transported to the CDC's National Center for Environmental Health for analysis. They used high-performance liquid chromatography-electrospray ionization-tandem mass spectrometry (HPLC–ESI–MS/MS) to quantitatively detect phthalate metabolites in urine. The NHANES Laboratory Procedures Manual (LPM) offers comprehensive guidelines for the collection and processing of specimens. In our investigation, we measured concentrations of ten specific phthalate metabolites, including mono(carboxynonyl) phthalate (MCNP), mono(carboxyoctyl) phthalate (MCOP), mono-2-ethyl-5-carboxypenty phthalate (MECPP), mono-n-butyl phthalate (MnBP), mono-(3-carboxypropyl) phthalate (MCPP), mono-ethyl phthalate (MEP), mono-(2-ethyl-5-hydroxyhexyl) phthalate (MEHHP), mono-isobutyl phthalate (MiBP), mono-(2-ethyl-5-oxohexyl) phthalate (MEOHP), and mono-benzyl phthalate (MBzP). Besides, mono-(2-ethyl)-hexyl phthalate (MEHP) and mono-isononyl phthalate (MiNP) were excluded because of the high percentage (> 20%) of values below the limit of detection (LOD). For values under the LOD, they were replaced with the LOD value divided by the square root of 2. The LOD for each phthalate metabolite varied slightly throughout the survey cycles and details are provided in Supplementary Table 1.

To reduce the bias in measurement inaccuracy brought on by urine dilution, investigators usually recommend standardizing measured urinary biomarker concentration based on the concentration of urinary creatinine [23]. We adopted a new method proposed by O'Brien et al. to adjust urinary phthalate metabolite concentrations [24]. We first created a model for urinary creatinine that took into account the covariates of age, sex, race/ethnicity, and BMI. The concentrations of adjusted phthalate metabolites were calculated by multiplying the measured metabolite concentration with the ratio of the predicted creatinine concentration to the actual observed creatinine concentration. All concentrations of phthalate metabolite shown in this article have been adjusted.

### Covariates

We identified several variables as potential confounders in our study: age, sex, race/ethnicity, education level, annual family income, smoking status, BMI status, hypertension, and diabetes mellitus (DM). For race/ethnicity, we categorized participants as non-Hispanic White, non-Hispanic Black, Mexican American, and other races. Education level was classified into three groups: less than high school, high school, and more than high school. Annual family income was stratified at the $20,000 mark, distinguishing between incomes under and over this threshold [25]. Smoking status was categorized as 'never' for individuals who have smoked fewer than 100 cigarettes in their lifetime, 'former' for past smokers with a history of over 100 cigarettes, and 'current' for those who have smoked more than 100 cigarettes in their lifetime and continue to smoke occasionally or daily [26]. BMI was calculated using weight in kilograms divided by height in meters squared, and participants were classified as underweight/normal (BMI < 25), overweight (25 ≤ BMI < 30), or obese (BMI ≥ 30).

### Statistical analysis

In our research, we expressed continuous variables as mean and standard error (SE) for those following a normal distribution. For data not adhering to a normal distribution, we used the median and quartiles for representation. Categorical variables were described using counts (n) and weighted percentages. Additionally, we utilized Pearson correlation coefficients to evaluate the relationship between each pair of log-transformed phthalate metabolite levels.

To investigate the association of natural log-transformed phthalate metabolites with periodontitis, we conducted weighted logistic regression analysis in three different models: a crude model without adjustment, Model 1 adjusted for age, sex, and race/ethnicity, and Model 2 further included adjustments for education level, annual family income, smoking status, BMI status, hypertension, DM, along with the covariates in Model 1. The results were expressed as odds ratios (ORs) with 95% confidence intervals (CIs). Additionally, we utilized restricted cubic spline (RCS) analysis to assess potential nonlinear relationships, setting knots at the 10th, 50th, and 90th percentiles of the phthalate metabolite concentrations. Furthermore, we categorized phthalate metabolites into quartiles to further analyze their association with periodontitis. A linear trend test (*P* for trend) was conducted by assigning median values of the quartiles to the phthalate metabolite variable treated as continuous.

We further investigated the consistency of the observed associations across different demographics, specifically age groups (< 60 and ≥ 60), genders, and race/ethnicity categories. In sensitivity analysis, we excluded pregnant women and cancer patients.

Given the minimal amount of missing data (< 5%), we opted for a complete case analysis approach. All analyses were adjusted for sample weights and performed using R software (version 4.3.1). We determined statistical significance based on *P* values being less than 0.05 in a two-tailed test.

## Results

### Study population

Within the 2009–2014 survey cycles, 10,683 participants, aged ≥ 30 years, underwent detailed periodontal assessments and possessed at least one natural tooth. We excluded participants who lacked complete information on phthalate metabolites (*N* = 7256) and body mass index (BMI) (*N* = 25). Finally, 3402 individuals were selected for inclusion in subsequent analysis, representing an estimated 141.71 million noninstitutionalized US adults aged ≥ 30 years. The weighted prevalence of total periodontitis, with its subcategories (mild, moderate, and severe), was found to be 42.37%, 4.61%, 30.63%, and 7.12% respectively. Compared with participants in the no/mild periodontitis group, those with moderate/severe periodontitis were more frequently older, men, with less education, low income, smokers, and those with hypertension and DM (Table 1). Supplementary Table 2 provides detailed characteristics of the study subjects based on the severity of periodontitis.

**Table 1:** Participant characteristics in NHANES 2009–2014 according to periodontitis status

Characteristic	Participants			
	**Overall** **(*n* = 3402)**	**Non-periodontitis** **(*n* = 1639)**	**Periodontitis** **(*n* = 1763)**	***P* value**
**Continuous variables, mean (SE)**				
Age	50.80(0.34)	48.19(0.48)	54.34(0.43)	< 0.001
Mean PPD	1.43(0.02)	1.15(0.02)	1.81(0.03)	< 0.001
Mean CAL	1.55(0.04)	1.08(0.02)	2.20(0.04)	< 0.001
**Categorical variables, n (weighted %)**				
Sex				< 0.001
Female	1688(50.94)	963(57.45)	725(42.08)	
Male	1714(49.06)	676(42.55)	1038(57.92)	
Race/ethnicity				< 0.001
Non-Hispanic White	1455(68.18)	825(74.61)	630(59.44)	
Non-Hispanic Black	696(10.59)	258(7.71)	438(14.49)	
Mexican American	478(8.06)	154(5.04)	324(12.17)	
Other	773(13.17)	402(12.64)	371(13.90)	
Educational level				< 0.001
Less than high school	814(15.92)	232(8.87)	582(25.53)	
High school	718(20.29)	287(17.09)	431(24.66)	
More than high school	1865(63.79)	1119(74.05)	746(49.81)	
Annual family income				< 0.001
Under $20,000	705(14.22)	233(9.38)	472(20.90)	
Over $20,000	2548(85.78)	1349(90.62)	1199(79.10)	
Smoking status				< 0.001
Never	1911(56.74)	1060(64.19)	851(46.59)	
Former	850(26.01)	380(24.77)	470(27.70)	
Now	640(17.25)	199(11.03)	441(25.72)	
BMI status				0.094
Underweight/normal weight	915(27.15)	465(28.74)	450(24.99)	
Overweight	1203(35.57)	578(35.80)	625(35.26)	
Obese	1284(37.28)	596(35.46)	688(39.75)	
Hypertension				< 0.001
No	1890(58.83)	1020(63.91)	870(51.92)	
Yes	1512(41.17)	619(36.09)	893(48.08)	
Diabetes mellitus				< 0.001
No	2898(88.11)	1465(90.81)	1433(84.46)	
Yes	481(11.89)	156(9.19)	325(15.54)	

*P* value by the t-test for continuous variables and the Chi-square test for categorical variables. Educational level: missing 5 values; Annual family income: missing 149 values; Smoking status: missing 1 value; Diabetes mellitus: missing 23 values

*NHANES* National Health and Nutrition Examination Survey, *PPD* Probing pocket depth, *CAL* Clinical attachment loss, *BMI* body mass index

Table 2 displays the median levels of urinary creatinine-corrected phthalate metabolites, along with their 25th and 75th quartiles, for both periodontitis and non-periodontitis groups. Notably, individuals with periodontitis exhibited lower levels of MCNP and MCOP, but higher levels of MECPP, MnBP, MEP, MEHHP, MEOHP, and MBzP when compared to those without periodontitis (*P* < 0.05). However, the levels of MCPP and MiBP did not show any statistical differences between the two groups (*P* > 0.05).

**Table 2:** The levels of phthalate metabolites in participants in NHANES 2009–2014 according to periodontitis status

Phthalate metabolites	Overall Median (Q25%, Q75%)	Non-periodontitis Median (Q25%, Q75%)	Periodontitis Median (Q25%, Q75%)	*P* value
MCNP, ng/mL	2.92(1.69,5.30)	3.04(1.76,5.82)	2.82(1.64,4.95)	0.021
MCOP, ng/mL	18.01(7.83,47.22)	19.71(8.52,51.75)	16.00(7.13,43.45)	< 0.001
MECPP, ng/mL	14.58(9.30,24.12)	13.88(8.74,23.03)	15.30(10.21,25.55)	0.008
MnBP, ng/mL	11.37(6.69,19.02)	10.69(6.24,18.11)	12.10(7.46,20.24)	< 0.001
MCPP, ng/mL	2.65(1.43,5.64)	2.66(1.38,5.74)	2.65(1.50,5.57)	0.784
MEP, ng/mL	47.15(19.73,136.00)	44.58(18.34,121.08)	52.08(21.31,169.92)	0.011
MEHHP, ng/mL	9.40(5.59,15.88)	8.85(5.03,15.25)	10.06(6.33,16.89)	< 0.001
MiBP, ng/mL	7.43(4.59,12.34)	7.21(4.59,12.06)	7.63(4.58,12.85)	0.065
MEOHP, ng/mL	5.95(3.61,9.48)	5.57(3.33, 9.12)	6.35(4.02,10.22)	0.001
MBzP, ng/mL	4.87(2.72,9.09)	4.59(2.51, 8.55)	5.26(3.07,10.07)	< 0.001

The concentrations of phthalate metabolites were standardized with covariate-adjusted creatinine based on the O’Brien et al. method. *P* value by the Mann–Whitney U test for continuous variables. Q25%, 25th percentile; Q75%, 75th percentile

### Correlations of phthalate metabolites

**Fig. 2:**
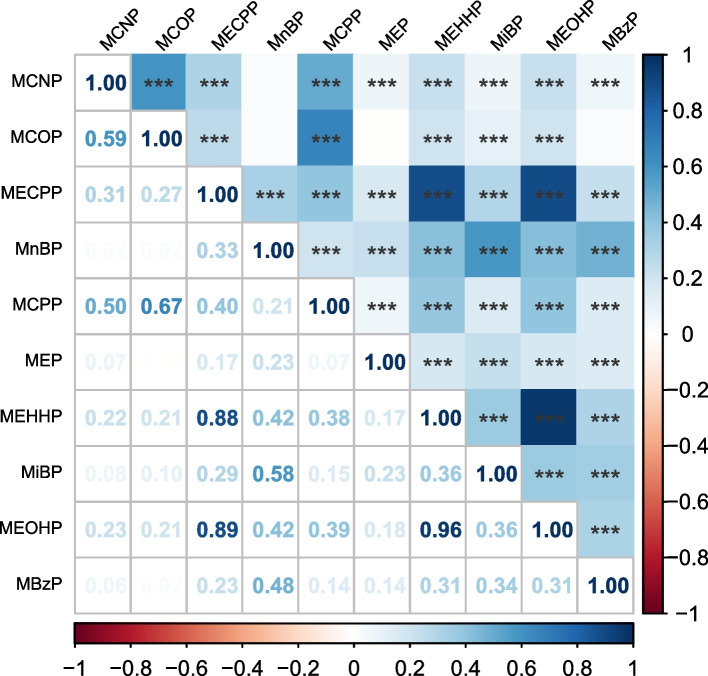
Pearson correlation heatmap of phthalate metabolites. The concentrations of phthalate metabolites were standardized with covariate-adjusted creatinine and log-transformed. *P < 0.05; **P < 0.01; ***P < 0.001

Figure 2 in our study illustrates the correlation coefficients among various phthalate metabolites, as determined through Pearson correlation analysis. Overall, the metabolites of phthalates demonstrated generally positive correlations with each other. Among these, the correlation between MEHHP and MEOHP was notably strong, registering a coefficient of *r* = 0.96. Additionally, there were also substantial correlations observed between MEOHP and MECPP (*r* = 0.89), and between MECPP and MEHHP (*r* = 0.88). This pattern of correlations suggests a significant degree of interrelation among these specific phthalate metabolites in our study population.

### Association of phthalate metabolites with periodontitis

Table 3 in our study presents the association of phthalate metabolites with periodontitis. In Model 2, which adjusted for all potential confounders, we observed that an increase of one standard deviation (SD) in the log-transformed levels of several phthalate metabolites was associated with higher odds of periodontitis. Specifically, the ORs were 1.08 (95% CI: 1.02–1.14) for MECPP, 1.07 (95% CI: 1.02–1.11) for MnBP, 1.10 (95% CI: 1.05–1.15) for MEHHP, 1.05 (95% CI: 1.01–1.09) for MiBP, 1.09 (95% CI: 1.04–1.14) for MEOHP, and 1.08 (95% CI: 1.03–1.13) for MBzP.

**Table 3:** OR (95% CI) summarizing the association between phthalate metabolites and periodontitis in participants in NHANES 2009–2014

Per-SD increase in log-transformed level		Quartiles of urinary phthalate metabolites concentrations (ng/mL)				
		Quartile 1	Quartile 2	Quartile 3	Quartile 4	*P* for trend
MCNP						
Crude model	0.86(0.78,0.95)*	1(reference)	0.90(0.66,1.22)	0.97(0.71,1.35)	0.75(0.57,0.98)*	0.02*
Model 1	0.90(0.80,1.01)	1(reference)	0.92(0.64,1.33)	1.06(0.72,1.56)	0.84(0.61,1.15)	0.19
Model 2	0.98(0.93,1.04)	1(reference)	1.01(0.85,1.19)	1.10(0.92,1.30)	1.00(0.86,1.16)	0.81
MCOP						
Crude model	0.84(0.76,0.92)***	1(reference)	0.86(0.64,1.16)	0.74(0.57,0.95)*	0.70(0.52,0.94)*	0.04*
Model 1	0.87(0.79,0.97)*	1(reference)	0.86(0.63,1.19)	0.81(0.61,1.07)	0.77(0.56,1.07)	0.2
Model 2	0.97(0.93,1.02)	1(reference)	0.94(0.82,1.09)	0.95(0.84,1.09)	0.97(0.84,1.12)	0.96
MECPP						
Crude model	1.16(1.04,1.30)*	1(reference)	1.55(1.16,2.09)**	1.41(1.01,1.95)*	1.73(1.26,2.37)**	0.01*
Model 1	1.16(1.03,1.30)*	1(reference)	1.66(1.18,2.34)**	1.49(1.05,2.12)*	1.68(1.21,2.33)**	0.02*
Model 2	1.08(1.02,1.14)*	1(reference)	1.29(1.10,1.52)**	1.23(1.05,1.45)*	1.32(1.13,1.55)**	0.01*
MnBP						
Crude model	1.19(1.08,1.30)***	1(reference)	1.34(1.07,1.69)*	1.43(1.16,1.77)**	1.56(1.18,2.05)**	0.01*
Model 1	1.17(1.07,1.28)***	1(reference)	1.38(1.07,1.77)*	1.41(1.15,1.74)**	1.55(1.17,2.05)**	0.01*
Model 2	1.07(1.02,1.11)**	1(reference)	1.15(1.00,1.31)*	1.18(1.06,1.33)**	1.20(1.05,1.38)*	0.02*
MCPP						
Crude model	0.99(0.90,1.09)	1(reference)	1.30(1.05,1.63)*	1.17(0.87,1.57)	1.12(0.86,1.47)	0.99
Model 1	1.03(0.92,1.15)	1(reference)	1.28(1.01,1.62)*	1.16(0.83,1.62)	1.22(0.91,1.64)	0.42
Model 2	1.03(0.97,1.08)	1(reference)	1.17(1.03,1.33)*	1.11(0.94,1.31)	1.14(0.97,1.33)	0.31
MEP						
Crude model	1.16(1.05,1.28)**	1(reference)	1.04(0.84,1.30)	1.13(0.88,1.45)	1.43(1.10,1.86)*	0.01*
Model 1	1.06(0.95,1.19)	1(reference)	0.95(0.75,1.21)	1.04(0.80,1.37)	1.13(0.84,1.52)	0.29
Model 2	1.00(0.96,1.05)	1(reference)	0.97(0.86,1.09)	1.00(0.88,1.14)	0.99(0.87,1.12)	0.96
MEHHP						
Crude model	1.20(1.09,1.33)***	1(reference)	1.41(1.10,1.79)*	1.43(1.05,1.93)*	1.71(1.28,2.28)***	0.001**
Model 1	1.21(1.08,1.34)***	1(reference)	1.39(1.05,1.83)*	1.53(1.09,2.15)*	1.66(1.21,2.30)**	0.005**
Model 2	1.10(1.05,1.15)***	1(reference)	1.18(1.04,1.34)*	1.24(1.07,1.45)*	1.30(1.11,1.52)**	0.003**
MiBP						
Crude model	1.11(1.02,1.21)*	1(reference)	1.01(0.79,1.28)	1.11(0.89,1.39)	1.21(0.92,1.60)	0.13
Model 1	1.13(1.04,1.24)**	1(reference)	1.10(0.82,1.48)	1.19(0.91,1.56)	1.25(0.93,1.68)	0.14
Model 2	1.05(1.01,1.09)*	1(reference)	1.02(0.88,1.19)	1.07(0.94,1.21)	1.07(0.93,1.23)	0.3
MEOHP						
Crude model	1.20(1.08,1.32)***	1(reference)	1.55(1.17,2.05)**	1.53(1.08,2.19)*	1.67(1.21,2.30)**	0.01*
Model 1	1.19(1.07,1.32)**	1(reference)	1.49(1.08,2.05)*	1.56(1.03,2.38)*	1.59(1.11,2.27)*	0.03*
Model 2	1.09(1.04,1.14)**	1(reference)	1.21(1.03,1.42)*	1.26(1.03,1.53)*	1.25(1.05,1.49)*	0.02*
MBzP						
Crude model	1.19(1.09,1.29)***	1(reference)	1.34(1.00,1.79)*	1.35(1.02,1.79)*	1.60(1.25,2.05)***	0.001**
Model 1	1.30(1.18,1.43)***	1(reference)	1.53(1.13,2.08)*	1.57(1.18,2.09)**	2.05(1.57,2.69)***	< 0.001***
Model 2	1.08(1.03,1.13)***	1(reference)	1.21(1.04,1.41)*	1.17(1.00,1.37)*	1.26(1.10,1.44)**	0.01*

The concentrations of urinary phthalate metabolites were standardized with covariate-adjusted creatinine based on the O’Brien et al. method. Crude model: unadjusted model. Model 1: adjusted for age, sex, and race/ethnicity. Model 2: adjusted for education level, income, smoking status, body mass index status, hypertension, diabetes mellitus, and covariates included in model 1. Test for trend (*P* for trend) was tested by incorporating the variables of the median of each quartile into the logistic regression model

*CI* Confidence interval, *NHANES* National Health and Nutrition Examination Survey, *SD* standard deviation

^*^P < 0.05

**P < 0.01

***P < 0.001

**Fig. 3:**
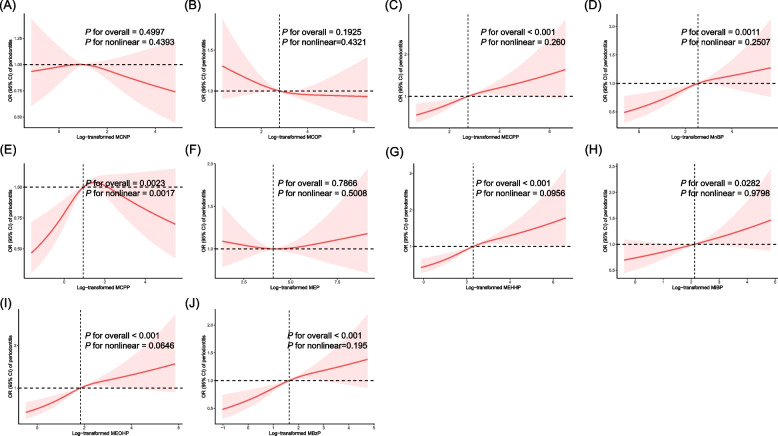
Restricted cubic splines of the relationship between urinary phthalate metabolites with periodontitis (**A**) MCNP. **B** MCOP. **C** MECPP. **D** MnBP. **E** MCPP. **F** MEP. **G** MEHHP. **H** MiBP. **I** MEOHP. **J** MBzP. ORs (red solid line) and 95% CIs (red shaded region) were calculated using the median log-transformed urinary phthalate metabolites levels as the reference value. The horizontal dashed line represented the reference OR of 1.0. All of the models were adjusted for age, sex, race/ethnicity, education level, income, smoking status, body mass index status, hypertension, and diabetes mellitus

Our RCS analysis revealed an inverted J-shaped relationship between log-transformed MCPP and periodontitis, with a statistically significant nonlinearity (*P* for nonlinear = 0.0017), as shown in Fig. 3. The inflection point for log-transformed MCPP was identified at 1.48 ng/mL.

When controlling for age, sex, and race/ethnicity, we observed notably higher ORs for periodontitis in individuals within the highest quartile of metabolite levels compared to those in the lowest quartile. The ORs were 1.68 (95% CI: 1.21–2.33, *P* for trend = 0.02) for MECPP, 1.55 (95% CI: 1.17–2.05,* P* for trend = 0.01) for MnBP, 1.66 (95% CI: 1.21–2.30, *P* for trend = 0.005) for MEHHP, 1.59 (95% CI: 1.11–2.27, *P* for trend = 0.03) for MEOHP, and 2.05 (95% CI: 1.57–2.69, *P* for trend < 0.001) for MBzP. However, further adjustment for factors in Model 2 slightly attenuated these associations, with ORs for MECPP, MnBP, MEHHP, MEOHP, and MBzP being 1.32 (95% CI: 1.13–1.55, *P* for trend = 0.01), 1.20 (95% CI: 1.05–1.38, *P* for trend = 0.02), 1.30 (95% CI: 1.11–1.52, *P* for trend = 0.003), 1.25 (95% CI: 1.05–1.49, *P* for trend = 0.02), and 1.26 (95% CI: 1.10–1.44, *P* for trend = 0.01), respectively.

### Subgroup analysis

In our subgroup analysis, we observed consistent effects across different demographics, including age, sex, and race/ethnicity, for the majority of the log-transformed phthalate metabolites. Specifically, the impacts of log-transformed MCNP, MCOP, MECPP, MnBP, MCPP, MEHHP, MiBP, MEOHP, and MBzP on periodontitis were uniform across these subgroups, as indicated by non-significant interaction terms (all *P* for interaction > 0.05), detailed in Supplementary Fig. 1. However, sex appeared to modify the relationship between log-transformed mono-ethyl phthalate (MEP) and periodontitis, as evidenced by a significant interaction term (*P* for interaction = 0.003).

Notably, the positive correlation between log-transformed MECPP, MnBP, MEHHP, MEOHP, and MBzP and periodontitis was apparent across all examined subgroups, underscoring the potential broad impact of these specific phthalate metabolites on periodontal health.

### Sensitivity analysis

We found similar relationships between phthalate metabolites and periodontitis in sensitivity analysis (Supplementary Table 3), proving the validity of our findings.

## Discussion

In this study, individuals with periodontitis exhibited higher levels of MECPP, MnBP, MEP, MEHHP, MEOHP, and MBzP when compared to those without periodontitis. Moreover, we observed a positive association between log-transformed MECPP, MnBP, MEHHP, MiBP, MEOHP, and MBzP and periodontitis, after adjusting for factors. It is important to note that the potential link between phthalate metabolites and periodontitis involves complex biological mechanisms that are not yet fully understood.

Research exploring the link between phthalates and periodontitis is limited. A previous study using NHANES data from 1999 to 2004 investigated the relationship between 156 environmental factors and periodontitis [13]. It reported that among current smokers, every one-point increase in the log-transformed level of mono-n-methyl phthalate was associated with a 47% increase in the likelihood of periodontitis (95% CI: 1.13–1.92) after adjusting for variables such as age, sex, race/ethnicity, socioeconomic status, and number of teeth. However, mono-n-methyl phthalate was included in the NHANES 2009–2010 and 2011–2012 cycles but was absent from the 2013–2014 cycle, and hence was not examined in our research. Instead, our study expanded the scope to include an additional 10 phthalate metabolites. By doing so, we conducted a more thorough analysis that encompassed multiple indicators, significantly enhancing the reliability of our findings. The observed pattern of consistent association between various phthalate metabolites and periodontitis underscores the potential biological effects these chemicals have in promoting such conditions. Given the ubiquity of phthalates in the environment, even a marginal increase in periodontitis risk could significantly affect public health. It is worth noting that the potential harm of phthalate metabolites may vary depending on factors such as individual differences, exposure time, pathways, and mixtures. As such, there is currently no clear consensus on safety thresholds for phthalate metabolites in urine.

Phthalates are categorized based on carbon chain length into low molecular weight (LMW) and high molecular weight (HMW) phthalates. MEHP, MECPP, MEHHP, and MEOHP are primary metabolites of di (2-ethylhexyl) phthalate (DEHP), a common HMW phthalate. phthalates are becoming increasingly prevalent due to their extensive application, thus elevating the risk of human exposure to these chemicals. The oral cavity, being a channel for both the respiratory and digestive systems, may be exposed to phthalates through various means. Phthalate metabolites have been detected in human saliva, though at lower levels than in serum and urine [27, 28].

Park et al. suggested that DEHP may reduce estrogen receptor alpha (ERα) activity [29]. Estrogens, as sex steroids, play a critical role in modulating the host immune response and maintaining bone homeostasis [30]. Notably, estrogen's influence extends beyond female bone turnover to encompass male bone metabolism as well [31, 32]. For instance, estrogen deficiency has been implicated in the onset of osteoporosis in elderly men [33]. Animal experiments have indicated that estrogen deficiency can exacerbate periodontal bone loss in rats with ligature-induced periodontitis [34, 35]. This leads us to speculate that phthalate metabolites might impact the progression of periodontitis by interfering with estrogen functions.

Previous research has indicated that chronic inflammation might act as a mediator in the link between Mono (2-ethylhexyl) phthalate (MEHP) exposure and the onset of depressive symptoms [36]. Specifically, the mediating effects of interleukin-6 (IL-6) and generalized inflammatory factors in this context were quantified as 15.96% (95% CI: 0.0288–0.1971) and 14.25% (95% CI: 0.0167–0.1899), respectively. In addition, another study has shown that dibutyl phthalate (DBP) can induce apoptosis in grass carp hepatocytes, mediated through mechanisms of inflammatory response and oxidative stress [14]. This highlights the broader biological impact of phthalates. In the field of periodontal health, the inflammatory response is intricately connected to the pathogenesis of periodontitis [15]. Maintaining a balance between the body’s defense system and oxidative stress is crucial in preserving periodontal tissue health [16]. The interplay of inflammatory reactions and oxidative stress, often resulting in mutual reinforcement, can lead to increased osteoclast activity and subsequent bone loss. This connection underlines the importance of understanding the complex interplay between environmental factors like phthalate exposure, oxidative stress, and inflammatory responses in the pathogenesis of periodontitis.

Phthalate metabolites have been shown to alter the composition of the gut microbiota in mice [37], which also could have implications for human health through the gut-brain axis [38]. Another study has observed variances in the respiratory tract microbiota among individuals exposed to different phthalate concentrations [39]. Although there are no studies directly linking phthalate metabolites to changes in oral microbiota, the well-established connection between the development of periodontitis and alterations in oral microbiota leads us to hypothesize that phthalate metabolites could influence periodontitis by affecting the microbiota.

In summary, phthalate metabolites may influence periodontitis through three main mechanisms: impacting estrogen function and affecting host inflammation as well as the structure of the microbiome. Future clinical prospective studies should investigate the role of individual biological components in periodontal disease. This includes a thorough examination of host immunity, epigenetic changes, and microbiome composition, all of which are critical to health and disease. By incorporating these variables, we can gain a better understanding of how environmental factors like phthalate exposure contribute to or mediate disease processes. This method will allow for a more thorough investigation of the complex interactions between genetic predisposition, microbial ecology, immune responses, and environmental influences in the development of periodontal disease.

However, it's crucial to recognize the limitations of our study. Firstly, due to its cross-sectional design, we cannot infer a causal link between phthalate metabolites and periodontitis. Secondly, despite adjusting for potential confounders based on prior studies and clinical knowledge, there may still be unmeasured or unknown confounding factors. Thirdly, differentiating the impacts of short-term versus long-term exposure to phthalate metabolites on periodontitis is challenging. Fourthly, the classification criteria for periodontitis used in this study do not allow for a diagnosis in patients with only one tooth. Furthermore, third molars were excluded from the periodontal assessments, potentially leading to an underestimation of the periodontitis prevalence. Lastly, the periodontal examination did not include individuals under 30 years old or those residing in institutional settings like nursing homes, hospitals, hospices, or prisons.

## Conclusion

Our study has identified a positive association of certain phthalate metabolites with periodontitis. These findings suggest a potential link between environmental exposures and periodontal health. However, conclusive findings regarding causality are not possible due to the nature of our study design. Therefore, future research should focus on tracking the long-term effects of phthalate metabolite exposure on periodontal health through prospective cohort studies. Additionally, advanced technologies including the microbiome, metabolomics, and proteomics analyses can be integrated with in vitro and in vivo experimental methods to elucidate the potential biological mechanisms linking phthalate exposure to periodontal disease. Only through this continued research can we fully understand the implications of phthalate exposure on oral health and potentially inform public health interventions and policy changes.

### Supplementary Information

Supplementary Material 1.

## Data Availability

The NHANES data was employed in this investigation, which can be found at https://wwwn.cdc.gov/nchs/nhanes/Default.aspx.
