# Supplementary material for: Association of phthalate metabolites with periodontitis: a population-based study
Source: BMC Oral Health. 2024 May 8;24:541. doi: 10.1186/s12903-024-04316-4 (PMC11080183; doi:10.1186/s12903-024-04316-4)
Supplement: Supplementary file 1 — Supplementary Material 1. [file 12903_2024_4316_MOESM1_ESM.docx]

Supplementary Table 1. The limit of detection for phthalate metabolites.

| **Phthalate metabolites (ng/mL)** | **Abbreviation** | **Limit of detection** | | |  |
| --- | --- | --- | --- | --- | --- |
|  |  | **NHANES 2009-2010** | **NHANES 2011-2012** | **NHANES 2013-2014** | |
| mono(carboxynonyl) phthalate | MCNP | 0.2 | 0.2 | 0.2 | |
| mono(carboxyoctyl) phthalate | MCOP | 0.2 | 0.2 | 0.3 | |
| mono-2-ethyl-5-carboxypenty phthalate | MECPP | 0.2 | 0.2 | 0.4 | |
| mono-n-butyl phthalate | MnBP | 0.4 | 0.4 | 0.4 | |
| mono-(3-carboxypropyl) phthalate | MCPP | 0.2 | 0.2 | 0.4 | |
| mono-ethyl phthalate | MEP | 0.462 | 0.6 | 1.2 | |
| mono-(2-ethyl-5-hydroxyhexyl) phthalate | MEHHP | 0.2 | 0.2 | 0.4 | |
| mono-isobutyl phthalate | MiBP | 0.2 | 0.2 | 0.8 | |
| mono-(2-ethyl-5-oxohexyl) phthalate | MEOHP | 0.2 | 0.2 | 0.2 | |
| mono-benzyl phthalate | MBzP | 0.216 | 0.3 | 0.3 | |

NHANES, National Health and Nutrition Examination Survey.

Supplementary Table 2. Participant characteristics according to periodontitis severity.

| **Characteristic** | **Participants** | | | |  |
| --- | --- | --- | --- | --- | --- |
|  | **Non-periodontitis (n=1639)** | **Mild periodontitis (n=154)** | **Moderate periodontitis (n=1244)** | **Severe periodontitis (n=365)** | ***P* value** |
| **Continuous variables, mean (SE)** | |  |  |  |  |
| Age | 48.19(0.48) | 47.68(1.41) | 55.52(0.48) | 53.57(0.84) | <0.001 |
| Mean PPD | 1.15(0.02) | 1.68(0.04) | 1.64(0.02) | 2.63(0.05) | <0.001 |
| Mean CAL | 1.08(0.02) | 1.24(0.04) | 2.03(0.04) | 3.53(0.12) | <0.001 |
| **Categorical variables, n (weighted %)** | |  |  |  |  |
| Sex |  |  |  |  | <0.001 |
| Female | 963(57.45) | 70(47.89) | 553(44.04) | 102(29.92) |  |
| Male | 676(42.55) | 84(52.11) | 691(55.96) | 263(70.08) |  |
| Race/ethnicity |  |  |  |  | <0.001 |
| Non-Hispanic White | 825(74.61) | 73(68.09) | 461(60.97) | 96(47.24) |  |
| Non-Hispanic Black | 258(7.71) | 32(12.88) | 280(12.55) | 126(23.88) |  |
| Mexican American | 154(5.04) | 26(8.43) | 218(11.93) | 80(15.62) |  |
| Other | 402(12.64) | 23(10.59) | 285(14.55) | 63(13.25) |  |
| Educational level |  |  |  |  | <0.001 |
| Less than high school | 232(8.87) | 34(12.78) | 389(24.46) | 159(38.37) |  |
| High school | 287(17.09) | 40(27.19) | 306(24.74) | 85(22.68) |  |
| More than high school | 1119(74.04) | 80(60.03) | 545(50.80) | 121(38.95) |  |
| Annual family income |  |  |  |  | <0.001 |
| Under $20,000 | 233(9.38) | 24(9.17) | 333(21.29) | 115(27.11) |  |
| Over $20,000 | 1349(90.62) | 125(90.83) | 849(78.71) | 225(72.89) |  |
| Smoking status |  |  |  |  | <0.001 |
| Never | 1060(64.20) | 94(60.32) | 623(47.14) | 134(35.35) |  |
| Former | 380(24.77) | 24(18.21) | 354(29.82) | 92(24.71) |  |
| Now | 199(11.03) | 36(21.47) | 266(23.04) | 139(39.95) |  |
| BMI status |  |  |  |  | 0.081 |
| Underweight/normal weight | 465(28.74) | 28(16.16) | 331(26.57) | 91(23.88) |  |
| Overweight | 578(35.80) | 50(35.21) | 441(35.01) | 134(36.36) |  |
| Obese | 596(35.46) | 76(48.63) | 472(38.42) | 140(39.76) |  |
| Hypertension |  |  |  |  | <0.001 |
| No | 1020(63.91) | 97(60.24) | 607(51.38) | 166(48.85) |  |
| Yes | 619(36.09) | 57(39.76) | 637(48.62) | 199(51.15) |  |
| Diabetes mellitus |  |  |  |  | <0.001 |
| No | 1465(90.81) | 137(86.46) | 992(83.42) | 304(87.60) |  |
| Yes | 156(9.19) | 17(13.54) | 247(16.58) | 61(12.40) |  |

*P* value by the t-test for continuous variables and the Chi-square test for categorical variables. PPD, probing pocket depth; CAL, clinical attachment loss; BMI, body mass index.

Supplementary Table 3. Sensitivity analysis after excluding pregnant women and cancer patients.

|  | Per-SD increase in log-transformed level | Quartiles of urinary phthalate metabolites concentrations (ng/mL) | | | |  |
| --- | --- | --- | --- | --- | --- | --- |
|  |  | Quantile 1 | Quantile 2 | Quantile 3 | Quantile 4 | *P* for trend |
| MCNP |  |  |  |  |  |  |
| Crude model | 0.84(0.76,0.93) *** | 1(reference) | 0.80(0.57,1.12) | 0.89(0.63,1.26) | 0.66(0.50,0.88) * | 0.005 ** |
| Model 1 | 0.87(0.78,0.98) * | 1(reference) | 0.80(0.54,1.17) | 0.96(0.63,1.46) | 0.74(0.53,1.03) | 0.08 |
| Model 2 | 0.97(0.91,1.02) | 1(reference) | 0.95(0.79,1.13) | 1.04(0.86,1.24) | 0.94(0.80,1.10) | 0.45 |
| MCOP |  |  |  |  |  |  |
| Crude model | 0.83(0.77,0.91) *** | 1(reference) | 0.81(0.63,1.06) | 0.73(0.57,0.93) * | 0.67(0.50,0.89) * | 0.02 * |
| Model 1 | 0.87(0.79,0.95) ** | 1(reference) | 0.81(0.60,1.08) | 0.78(0.59,1.04) | 0.73(0.54,1.00) * | 0.15 |
| Model 2 | 0.97(0.93,1.01) | 1(reference) | 0.93(0.82,1.05) | 0.95(0.84,1.09) | 0.96(0.83,1.10) | 0.86 |
| MECPP |  |  |  |  |  |  |
| Crude model | 1.12(1.02,1.24) * | 1(reference) | 1.42(1.10,1.84) * | 1.31(0.98,1.76) | 1.57(1.20,2.05) ** | 0.01 * |
| Model 1 | 1.12(1.01,1.24) * | 1(reference) | 1.49(1.11,2.00) * | 1.37(1.01,1.87) * | 1.53(1.16,2.02) ** | 0.03 * |
| Model 2 | 1.06(1.01,1.11) * | 1(reference) | 1.20(1.05,1.37) * | 1.17(1.03,1.34) * | 1.24(1.09,1.42) ** | 0.02 * |
| MnBP |  |  |  |  |  |  |
| Crude model | 1.16(1.06,1.27) ** | 1(reference) | 1.47(1.15,1.88) ** | 1.39(1.13,1.73) ** | 1.53(1.14,2.05) * | 0.02 * |
| Model 1 | 1.16(1.07,1.27) ** | 1(reference) | 1.46(1.11,1.91) * | 1.39(1.10,1.75) * | 1.54(1.15,2.06) ** | 0.01 * |
| Model 2 | 1.06(1.01,1.11) * | 1(reference) | 1.18(1.01,1.37) * | 1.16(1.02,1.33) * | 1.19(1.02,1.39) * | 0.06 |
| MCPP |  |  |  |  |  |  |
| Crude model | 0.98(0.90,1.07) | 1(reference) | 1.33(1.06,1.66) * | 1.17(0.90,1.50) | 1.13(0.87,1.46) | 0.99 |
| Model 1 | 1.01(0.91,1.12) | 1(reference) | 1.27(1.01,1.60) * | 1.12(0.85,1.48) | 1.22(0.92,1.60) | 0.42 |
| Model 2 | 1.02(0.97,1.07) | 1(reference) | 1.17(1.03,1.32) * | 1.08(0.94,1.25) | 1.13(0.97,1.31) | 0.36 |
| MEP |  |  |  |  |  |  |
| Crude model | 1.17(1.06,1.30) ** | 1(reference) | 0.97(0.77,1.22) | 1.15(0.88,1.50) | 1.40(1.06,1.85) * | 0.01 * |
| Model 1 | 1.08(0.97,1.20) | 1(reference) | 0.91(0.71,1.17) | 1.06(0.80,1.42) | 1.11(0.82,1.50) | 0.32 |
| Model 2 | 1.00(0.96,1.05) | 1(reference) | 0.94(0.83,1.07) | 1.00(0.87,1.15) | 0.96(0.84,1.10) | 0.76 |
| MEHHP |  |  |  |  |  |  |
| Crude model | 1.18(1.08,1.29) *** | 1(reference) | 1.43(1.15,1.77) ** | 1.30(0.97,1.73) | 1.67(1.29,2.16) *** | 0.001 *** |
| Model 1 | 1.18(1.07,1.30) ** | 1(reference) | 1.39(1.08,1.80) * | 1.36(0.98,1.87) | 1.59(1.20,2.13) ** | 0.01 * |
| Model 2 | 1.09(1.04,1.13) *** | 1(reference) | 1.16(1.03,1.31) * | 1.17(1.01,1.34) * | 1.26(1.10,1.45) ** | 0.004 ** |
| MiBP |  |  |  |  |  |  |
| Crude model | 1.11(1.01,1.22) * | 1(reference) | 0.99(0.77,1.28) | 1.14(0.88,1.49) | 1.18(0.88,1.59) | 0.21 |
| Model 1 | 1.13(1.03,1.25) * | 1(reference) | 1.08(0.78,1.49) | 1.23(0.92,1.64) | 1.20(0.86,1.66) | 0.28 |
| Model 2 | 1.04(1.00,1.09) | 1(reference) | 1.00(0.85,1.18) | 1.07(0.92,1.24) | 1.04(0.89,1.22) | 0.54 |
| MEOHP |  |  |  |  |  |  |
| Crude model | 1.17(1.07,1.28) *** | 1(reference) | 1.40(1.10,1.77) * | 1.35(0.96,1.89) | 1.60(1.21,2.12) ** | 0.01 * |
| Model 1 | 1.16(1.06,1.28) ** | 1(reference) | 1.31(0.98,1.76) | 1.35(0.91,2.01) | 1.52(1.11,2.08) * | 0.02 * |
| Model 2 | 1.08(1.03,1.12) ** | 1(reference) | 1.12(0.98,1.29) | 1.16(0.97,1.38) | 1.21(1.04,1.41) * | 0.02 * |
| MBzP |  |  |  |  |  |  |
| Crude model | 1.17(1.08,1.28) *** | 1(reference) | 1.47(1.09,1.97) * | 1.36(1.01,1.83) * | 1.65(1.27,2.14) *** | 0.001 ** |
| Model 1 | 1.28(1.15,1.42) *** | 1(reference) | 1.73(1.26,2.37) ** | 1.60(1.18,2.17) ** | 2.10(1.55,2.83) *** | <0.001*** |
| Model 2 | 1.06(1.01,1.12) * | 1(reference) | 1.28(1.08,1.51) * | 1.18(0.99,1.40) | 1.26(1.08,1.47) * | 0.04 * |

The concentrations of phthalate metabolites were standardized with covariate-adjusted creatinine based on the O’Brien et al. method. Crude model: unadjusted model. Model 1: adjusted for age, sex, and race/ethnicity. Model 2: adjusted for education level, income, smoking status, body mass index status, hypertension, diabetes mellitus, and covariates included in model 1. Test for trend (*P* for trend) was tested by incorporating the variables of the median of each quartile into the logistic regression model. CI, confidence interval; NHANES, National Health and Nutrition Examination Survey; SD, standard deviation. **P* < 0.05; ***P* < 0.01; ****P* < 0.001.


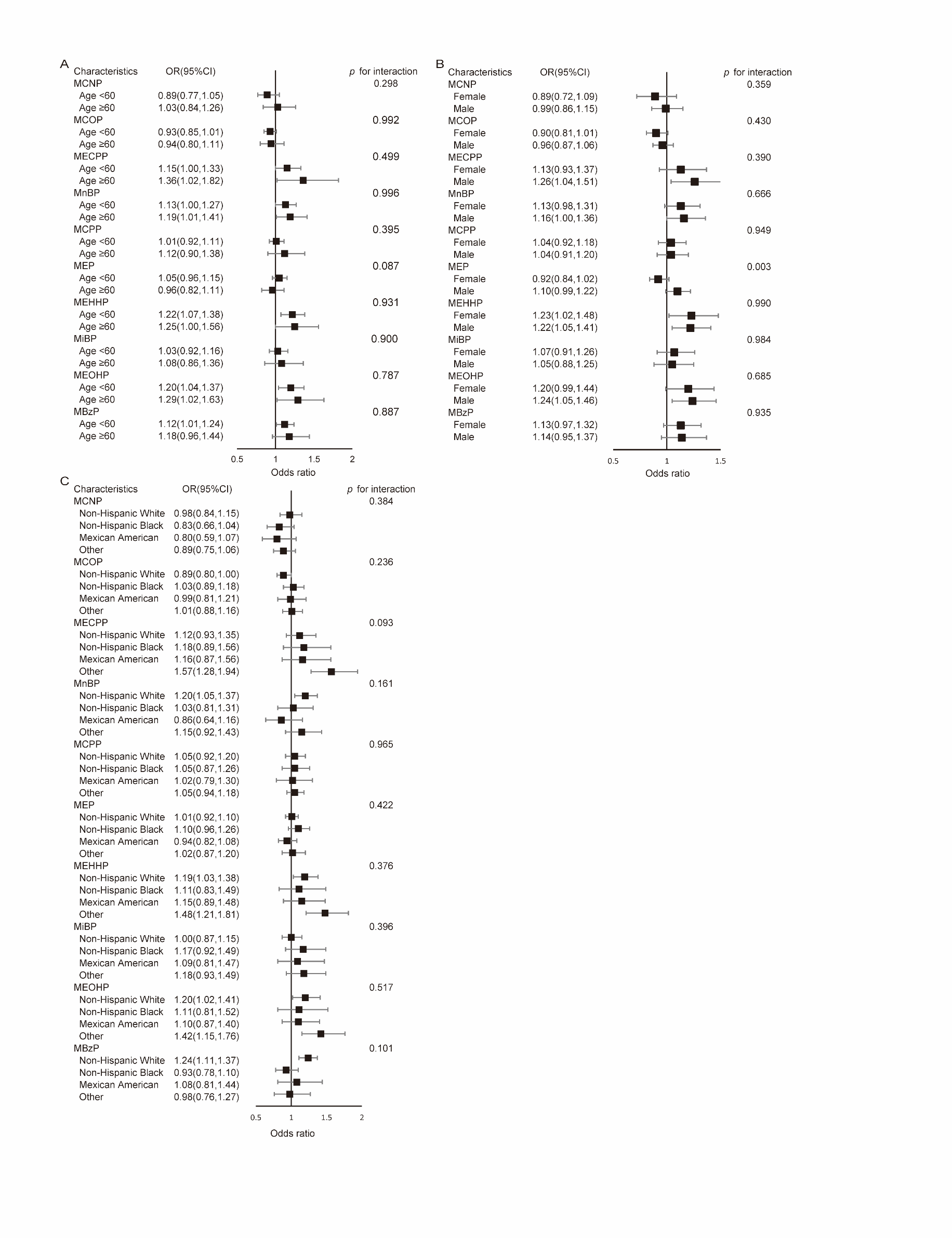


Supplementary Figure 1. Subgroup analysis of the associations between phthalate metabolites and periodontitis in participants (A) age. (B) sex. (C) race/ethnicity.

The concentrations of phthalate metabolites were standardized with covariate-adjusted creatinine and log-transformed. OR estimates were adjusted for age, sex, race/ethnicity, education level, income, smoking status, body mass index status, hypertension, and diabetes mellitus unless the covariate was tested as an interaction term. OR, odds ratio; CI, confidence interval; BMI, body mass index.
